# Safety and efficacy of physiologist-led dobutamine stress echocardiography: experience from a tertiary cardiac centre

**DOI:** 10.1530/ERP-18-0038

**Published:** 2018-07-02

**Authors:** Theodoros Ntoskas, Farhanda Ahmad, Paul Woodmansey

**Affiliations:** Department of Cardiology, Heart and Lung Centre, New Cross Hospital, Wolverhampton, UK

**Keywords:** stress echocardiography, cardiac physiologist, dobutamine, coronary artery disease, safety

## Abstract

**Background:**

Dobutamine stress echocardiography (DSE) services have traditionally been medically led. In some UK institutions, DSE lists are led by physiologists with medical support. In our tertiary cardiac centre at New Cross Hospital (NCH), the DSE service was established by a consultant echocardiographer. Following intensive training and assessment, the Trust approved drug administration by named senior cardiac physiologists. We believe this is the first report of a cardiac physiologist-managed DSE service, including physiologist drug administration. We have assessed the feasibility, safety and validity of this physiologist-led DSE service.

**Methods:**

Retrospective analysis of 333 patients undergoing stress echocardiogram for inducible reversible ischaemia, myocardial viability and valvular heart disease over 6 months. Patients’ case notes review after 18–24 months.

**Results:**

Overall, 92% of all cases (306) were performed by physiologists. In 300 studies, dobutamine was administered. The majority of the referrals were for coronary artery disease (CAD) assessment (281). In 235 cases, the study was uncomplicated. Sixty-seven patients developed dobutamine-related side effects. In 16 cases, complications led to early termination of the study. In two cases, urgent medical review was needed. Of the 281 studies for CAD assessment, 239 were negative for ischaemia, 28 were positive and 14 inconclusive. In 5 out of 28 cases with echocardiogram, evidence of inducible ischaemia, coronary angiography revealed unobstructed coronary arteries.

**Conclusion:**

This study demonstrates the safety and effectiveness of this practice and provides potential for the expansion of the physiologists’ role and physiologist-led DSE services in other hospitals.

## Introduction

Stress echocardiography (SE) is a well-established, reliable and safe method for assessment of ischaemic heart disease (1). Furthermore, SE is utilised in patients with valvular heart disease or cardiomyopathies and is also used for determining the extent of hibernating myocardium (2). The stressor used for SE can be physical exercise, pacemaker stress, pharmacological agent or a combination of pharmacological agents (3). Dobutamine is the preferred pharmacological stressor (4). There are a number of hospitals in the United Kingdom, where physiologists lead safe and effective SE services especially in the evaluation of CAD (5, 6). However, to our knowledge, in these other centres dobutamine and other intravenous drugs are administered by a nurse or doctor. The SE service in our centre was initially established in 2009 by a consultant echocardiographer, who is also a registered cardiac physiologist, with support from the consultant cardiologists and with drug administration by a cardiology registrar. To reduce the waiting time and meet the demand for the expansion of the SE service, and acknowledging the skills, expertise and the extended role of the physiologists (7); a physiologist-led dobutamine stress echocardiography (PLDSE) service, including physiologist drug administration, was established in our tertiary cardiac centre by August 2015. We sought to assess the effectiveness and the safety of PLDSE service in real-life practice and compare these measures with existing literature.

## Materials and methods

### Trust policy

Cardiac physiologists are non-state registered members of the multidisciplinary ‘Heart Team’ (8). Many are members of the Registration Council of Clinical Physiologists (RCCP) and the British Society of Echocardiography (BSE). They have clearly defined and uniquely specialised roles in cardiac diagnostics including responsibility for all aspects of patient care associated with stress testing.

Currently, cardiac physiologists are not regulated by statute and therefore cannot be considered for exemptions under the medicines legislation (9). To aid best practice and compliance, cardiac physiologists can only legally be involved in prescribing via a patient-specific direction, which is a written instruction or prescription given by an independent prescriber to another professional to administer a medicine to a specific patient (10).

Protocols were developed for drugs routinely given by the cardiac physiologist during the stress test within the dose range under the direct written instruction from an appropriate prescriber and for additional drugs, which may be needed in response to changes in the patient’s clinical condition. The agreed drug administration protocols were endorsed by the clinical lead for the Cardiology Directorate under the auspices of the Cardiology Governance Committee (11). The procedure was further ratified by the Medicines Management Committee and a change was made to the Trust’s medicine management policy to permit cardiac physiologists to administer prescribed medication orally and intravenously. The protocols were reviewed and updated accordingly. A pre-printed prescription proforma of all the drugs, including dose ranges, that could be administered as and when needed during the stress echo was introduced to facilitate the service (Supplementary Table 1, see section on supplementary data given at the end of this article). The physician who assessed the patient for the DSE also completed and signed the prescription and sent it with the referral to the DSE service. Cardiac physiologists involved in drug administration attended and completed the trust intravenous therapy course. This provided theoretical knowledge and practical skills in the safe practice of intravenous and injection therapy, guided by national guidelines and Trust policies. Training included a written exam, practical assessments and a log book. Cannulation training was mandatory to ensure that cardiac physiologists were supported to undertake the responsibilities outlined. Life support training was essential for the team undertaking the stress echo. At least one member was trained in advanced life support and the other in immediate life support. Medical cover arrangements were put in place where a named doctor was aware of the patients on the stress echo list and was contactable should urgent advice be needed or should the clinical condition of the patient deteriorate. The doctor was usually available in the near vicinity and was required to sign a form with contactable details and attend immediately if needed (Supplementary data 2).

### Sampling frame

Consecutive patients who underwent elective outpatient DSE for the assessment of CAD, the evaluation of myocardial viability or the significance of aortic valve stenosis during the period of 18th August 2015 to 17th February 2016 were included in this service review. The patients were referred by cardiologists, other speciality physicians or chest pain nurse specialists. At the time of data collection for this study, the National Institute for Health and Care Excellence (NICE) guidelines recommended SE as a non-invasive functional imaging investigation for diagnosing myocardial ischaemia in people with a moderate pre-test likelihood that chest pain was caused by angina (30–60%) and an uncertain diagnosis (12).

### Data collection

Patient demographics, relevant medical history, cardiac medication, stress echocardiogram report, other functional non-invasive tests findings, including cardiac MRI, CT, coronary angiogram or myocardial perfusion scintigraphy and invasive coronary angiogram data were reviewed using hospital electronic databases retrospectively.

The following data were collected from the DSE studies and reports: reason for the study, dobutamine-related complications, baseline left ventricle (LV) systolic function and evidence of reversible inducible ischaemia in patients referred for CAD assessment.

### Ethics statement

In accordance with UK guidance from the National Research and Ethics Service, this study was registered with our National Health Service (NHS) Trust as a service evaluation for which local institutional approval was sought and obtained. Additionally, it was confirmed that patient consent and ethical approval were not required.

### Physiologist-led DSE

A cardiac physiologist-led SE service was started in August 2015 in New Cross Hospital (NCH). Currently, the service complies with the updated regulations of setting up a stress echo service (13) with the implementation of the aforementioned local drug administration policies during SE. The PLDSE team consists of two experienced transthoracic BSE accredited and RCCP registered cardiac physiologists. One of them is responsible for leadership, image acquisition, interpretation and reporting of the study and the other for monitoring the heart rhythm, blood pressure and the administration of all relevant medication.

### Dobutamine stress protocols/imaging protocols

**Figure 1:**
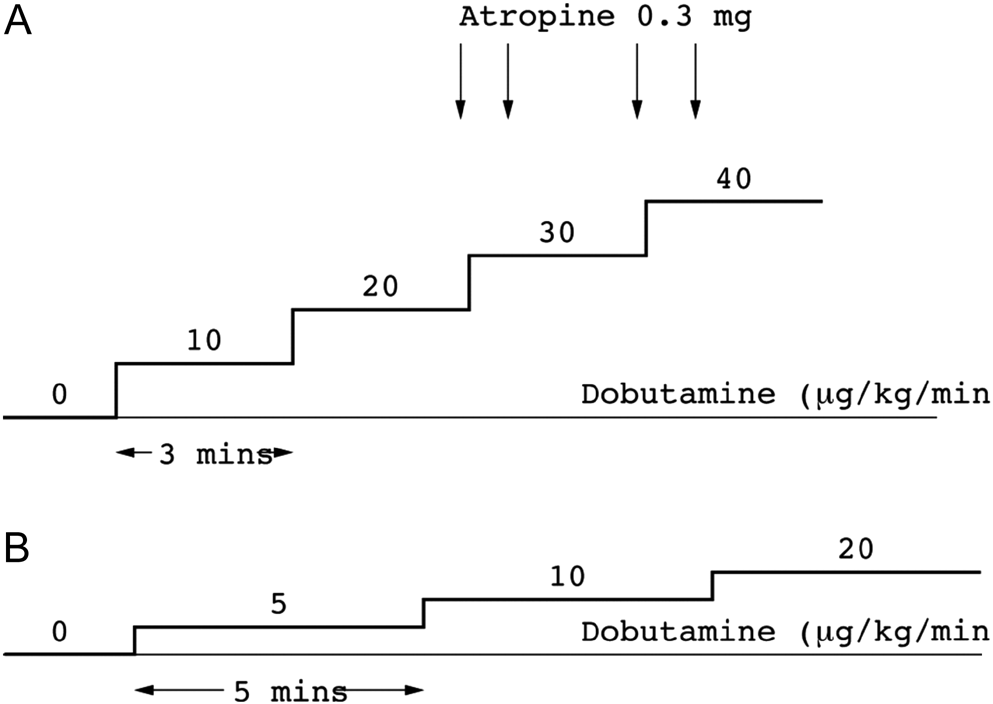
Dobutamine stress protocols. (A) Protocol A. (B) Protocol B.

PLDSE utilises two dobutamine stress protocols, the full dobutamine protocol for assessment of myocardial ischaemia (protocol A) and a low-dose protocol for the assessment of myocardial viability/LV contractile reserve in aortic stenosis (protocol B). These protocols are in accordance with the BSE procedure guidelines for the clinical application of SE. Atropine, when required to augment the heart rate response, is also helpful in reducing the likelihood of vagal reactions (Fig. 1).

Imaging was performed using a standard protocol for rest and peak stress acquisition visualising the 16 American Heart Association LV myocardial segments (14). For aortic stenosis assessment, additional imaging views and Doppler measurements were needed (15).

### Contrast use

In addition, all the protocols allowed the use of contrast for LV opacification when imaging windows were suboptimal. Contrast helped to improve endocardial delineation when two or more continuous myocardial segments were not well visualised on standard harmonic imaging. Following the European Society of Cardiology recommendations, contrast was used to enhance detection of regional wall-motion abnormalities and could also be used to assess myocardial perfusion (16). In this study, bolus contrast using a specific pre-set on the ultrasound system was used to enhance the endocardial borders.

### Risks and contraindications for DSE

Absolute contraindications for DSE include previous hypersensitivity/allergy to dobutamine, recent myocardial infarction, ongoing unstable angina, acute heart failure, LV thrombus, recent ventricular arrhythmias, uncontrolled supraventricular arrhythmias, high grade atrioventricular block, severe dynamic or fixed LV outflow tract obstruction, severe uncontrolled hypertension and active endocarditis or myopericarditis. A poor acoustic window makes any form of SE unfeasible to perform. However, this limitation of SE today should not exceed 5% of all referrals. With new transducer technology using harmonic imaging and the use of intravenous contrast agents for LV opacification, optimal endocardial border delineation is achievable in the vast majority of patients and should be available in every stress echocardiogram laboratory (13). Atropine is generally contraindicated in patients with closed-angle glaucoma and severe prostatic disease.

Before each study was undertaken, the patient was sent an appointment letter and a consent information leaflet explaining the procedure together with the potential risks and benefits. Consent was confirmed on the day of the test. The following risks were quoted: 1 in 2000 risk of life-threatening events including ventricular arrhythmias and myocardial infarction and 1 in 10,000 risk of death or allergic potentially life-threatening reaction to drugs and contrast. These rates derive from literature and echocardiography department experience (17).

### Classification and clinical implications of SE response

Visual assessment of endocardial excursion and wall thickening was used for analysis of stress echocardiograms. Function in each segment was graded at rest and with stress as normal, hyperdynamic, hypokinetic, akinetic, dyskinetic or aneurysmal. Images from low or intermediate stages of dobutamine infusion were compared with peak stress images to maximise the sensitivity for the detection of coronary disease (1). A normal stress echocardiogram result was defined as normal LV wall motion at rest and with stress. Resting wall-motion abnormalities, unchanged with stress, were classified as ‘fixed’ and most often represented regions of prior infarction. Patients with fixed wall-motion abnormalities and no inducible ischaemia were not considered as having a normal study result. Abnormal study findings included those with fixed wall-motion abnormalities or new or worsening abnormalities indicative of ischaemia. In addition to the evaluation of segmental function, the global LV response to stress should be assessed. Stress-induced changes in LV shape, cavity size and global contractility have been shown to indicate the presence or absence of ischaemia (18).

### Dobutamine adverse events definitions

Major arrhythmic events were defined as life-threatening rhythmic complications (cardiac asystole, advanced atrioventricular block, ventricular fibrillation or sustained ventricular tachycardia) or complications that required hospital admission. Arrhythmic events which led to early termination of the test, but required no other action were not considered major events.

Minor arrhythmic events were defined as the development of uniform or multiform premature ventricular beats, ventricular bigeminy or couplets, short episodes of self-terminating supraventricular tachycardia and non-sustained ventricular tachycardia.

Severe hypotension was defined by an arterial pressure drop  ≥ 40 mmHg with symptoms.

Non-cardiac side effects were defined as the development of headache, dizziness, nausea and vomiting.

## Results

### SE service at New Cross Hospital

During the 6-month audit period, 333 patients attended the echocardiography department for an elective stress echocardiogram. Of these, 306 (91.9%) SEs were physiologist-led studies. In two cases, the study was not performed, one due to very limited apical echo windows and one because of the patient’s severe anxiety. As this article is about the effectiveness and safety of the PLDSE and because the overwhelming majority of the studies were performed by physiologists, the following data analysis and discussion focuses on the outcomes of the tests performed by the cardiac physiologist team.

### Physiologist-led DSE services at NCH

Dobutamine was the commonly used stressor in our hospital. Three hundred (98%) out of 306 tests were pharmacological stress echocardiograms. The majority of DSE requests were to investigate possible reversible inducible ischaemia due to CAD (281, 93.7%) in accordance with the NICE guidelines (12). The remainder of the DSE requests were to clarify the severity of aortic valve stenosis in patients with significantly impaired LV systolic function and low flow and low gradient aortic valve stenosis (19). Low-dose stress echo in this group was to determine the left ventricular contractile reserve and extent of viable myocardium in order to inform a clinical decision regarding the need for revascularisation in patients with known CAD (Table 1).

**Table 1:** Indications for DSE.

	Physiologist-led DSE (*n* = 300)
Inducible reversible ischaemia	281 (93.7%)
Valve assessment/contractile reserve	14 (4.7%)
Viability	5 (1.6%)

DSE, dobutamine stress echocardiography.

### Demographics

The average age of the patients enrolled in this service review was 60.8 years. The youngest patient was a 26-year-old woman and the oldest a 90-year-old man both referred for chest pain assessment. The majority of patients were male (57%). Sixty-five percent of the total were referred by cardiology consultants or registrars from the outpatient clinic 15% of the referrals came from chest pain specialist nurses from the rapid access angina clinic or following assessment of in-patients. In addition, a number of DSE requests came from the driver and vehicle licensing agency for assessment of the eligibility of the patient to hold a driver’s licence. A small number of referrals were from other clinicians as part of assessment before non-cardiac operations or organ transplant.

### DSE interpretation results

Overall, 281 of the 300 DSEs were performed for the assessment of ischaemic heart disease or to rule out CAD. Of these, 239 studies were negative for reversible inducible ischaemia, 28 were positive and 14 inconclusive.

Regarding the inconclusive tests, in two cases, the targeted heart rate for the individual patient was not achieved despite the maximum dose of dobutamine and the administration of atropine. Significant side effects related to dobutamine administration were observed in 11 cases, forcing early termination of the test. Only one patient’s DSE was cancelled due to very poor apical echocardiography images.

Contrast for LV opacification was used in 267 DSEs (89%).

Of the 28 patients who had a positive DSE for reversible inducible ischaemia, 26 underwent coronary angiography and 2 were treated medically for stable angina without any further invasive investigations. In 21 cases (80.8%), the angiogram confirmed the DSE findings with significant CAD in one, two or three vessels. In five cases (19.2%), the diagnostic angiogram revealed unobstructed epicardial coronary arteries.

Eight of 239 patients (3.3%) with negative DSE for ischaemia, subsequently had a coronary angiogram, either because they were admitted with an acute coronary syndrome or presented with recurrent anginal symptoms, or an angiogram was performed before a valve operation. All eight showed severe coronary disease (Table 2).

**Table 2:** False negative DSE studies.

Age (gender)	DSE was performed for	Angiogram was performed for	Elapsed time between DSE and angiogram (months)	Angiogram findings	Outcome
54 (M)	Ischaemia	Out of hospital arrest	7	Significant 3VD	CABG
74 (M)	Ischaemia	ACS (NSTEMI)	12	Significant LMS and 3VD disease	MDT decision for medical and device treatment due to severe LVSD
67 (F)	Ischaemia – previous CABG	ACS (NSTEMI)	16	Severe RCA stenosis	PCI to RCA
45 (M)	Ischaemia	Exertional angina	10	Significant 2VD	CABG
65 (F)	Ischaemia	Exertional angina	22	Significant 3VD	CABG
61 (M)	Ischaemia	Exertional angina	22	Significant 3VD	CABG
75 (F)	Ischaemia	Prior mitral and tricuspid valve operation	16	Significant mid LAD and ostial LCx disease	CABG and mitral and tricuspid valve repair
73 (M)	Severity of AS-ischaemia	Prior aortic valve operation	2	Significant 3VD	CABG + aortic valve replacement

ACS, acute coronary syndrome; AS, aortic stenosis; CABG, coronary artery bypass graft; DSE, dobutamine stress echocardiogram; F, female; LAD, left anterior descending artery; LCx, left circumflex; LMS, left main stem; LVSD, left ventricle systolic dysfunction; M, male; MDT, multidisciplinary team; NSTEMI, non-ST elevation myocardial infarction; RCA, right coronary artery; VD, coronary vessel disease.

The clear majority (231) of the patients who had a negative DSE for ischaemia were discharged back to their general practitioner (GP). In this group, none were referred for recurrent episodes of chest pain by their GP, nor attended the emergency department for typical cardiac chest pain. This group did not have any further invasive or non-invasive testing for the detection of CAD within the time period of 18–24 months after the performed DSE, as per the information derived by the clinical web portal of NCH. We cannot rule out attendances at other institutions.

### DSE safety

Overall, 235 DSE studies (78.6%) were uncomplicated with no recorded dobutamine or atropine side effects.

A major arrhythmic event was observed in one patient (0.3%), a 39-year-old woman with no previous history of cardiac disease and limited mobility, referred for CAD assessment. She developed a severe vasovagal episode with profound bradycardia and junctional rhythm, which resolved with the administration of 600 µg of atropine. Her LV function was normal and the DSE was negative.

Minor arrhythmic events, resulted in the early termination of DSE in five cases (1.7%). These dobutamine-induced arrhythmic complications included isolated ventricular ectopics, bigeminy, couplets, triplets or short runs of non-sustained ventricular tachycardia. These arrhythmias occurred in male patients with known ischaemic heart disease and in two cases severely impaired LV systolic function. In all cases, the arrhythmia terminated after the discontinuation of dobutamine infusion.

Minor rhythmic complications were registered in 14.1% (42 patients). All minor arrhythmic complications were well tolerated.

Dobutamine infusion provoked severe systolic blood pressure fall in 5 (1.7%) subjects, and this was usually at the higher dose of 40 µg/kg/min. One patient suffered from a severe vasovagal episode and significant blood pressure drop during cannulation.

Non-cardiac side effects appeared in 7 (2.4%) subjects. In two of these episodes, the intensity of the symptoms prompted early termination of the dobutamine administration and target heart rate was not achieved, and thus the study was inconclusive.

Two studies were terminated early because the patient became severely symptomatic with severe chest pain or shortness of breath, respectively. In one case, the patient developed dobutamine-induced hyperdynamic LV response, which led to LV systolic cavity obliteration and a large increase in LV outflow track gradient accompanied by a significant blood pressure drop.

In two events, it was necessary for the cardiology registrar to intervene and support the physiologist team. Both of these incidences were due to severe and prolonged vasovagal episodes, which required fluid resuscitation.

In this review, no deaths, cardiac rupture or myocardial infarction were registered.

## Discussion

SE services have traditionally been led by cardiologists with physiologist and nurse support, particularly in the setting of dobutamine stress.

To our knowledge, this is the first data analysis of the effectiveness and safety of an independent cardiac physiologist-managed DSE service, including the administration of intravenous drugs and the performance and reporting of the stress echo study. In this context, the cardiac physiologist has the responsibility not only to perform and interpret the findings of the study but also to deal with any complication that may arise in a timely, safe and effective manner. The development of highly trained and specialised cardiac physiologists and the emergence of the new consultant echocardiographer role (20), accommodated by the National Higher Specialist Scientific Training programme (21), has the potential to accelerate the growth of independent physiologist-led services within the NHS of England.

### Limitations

This paper is a service review, which focused on the unique and innovative UK-based cardiac physiologist-managed DSE service, including administration of intravenous medication as well as reporting the study. The agreement between PLDSE and invasive coronary angiography as a gold standard of accuracy was not assessed, so the precise diagnostic accuracy of the PLDSE could not be evaluated especially for the negative DSE studies (specificity).

In addition, this is a retrospective analysis and event data were obtained from the clinical web portal of the Trust, so we do not have any data regarding cardiac events, which might have occurred in our patients who did not present to this Trust.

### Quality assurance of PLDSE service

The coronary angiographic cut-off for luminal diameter stenosis at which wall-thickening abnormalities occur is 58% for DSE (22). The reported sensitivity for the detection of CAD (cut off of >50% luminal diameter stenosis) is approximately 80% with a specificity of approximately 86% for dobutamine stress results (1, 23, 24). However, it is clear that the diagnostic accuracy of any test varies according to the pre-test likelihood of CAD in the population tested. In this study, all the patients apart from two (26 in total) who had evidence of reversible ischaemia on DSE underwent coronary angiograms. Of these, 80.8% (21 patients) had flow-limiting CAD, which is in keeping with the literature base.

Our practice involves discussion and reporting of challenging cases at both the weekly echo multidisciplinary team (MDT) meeting and at the cardiothoracic MDT meeting. Feedback is also given by the intervention specialists for discrepancies noted between coronary angiogram and SE data. This is part of the ongoing learning and improving of service quality standards.

In the interest of service quality, all the false-positive and false-negative cases in this study were discussed, and the images reviewed at a dedicated discrepancy MDT meeting. From the eight false-negative cases, minor amendments have been agreed in two reports regarding the assessment and grade of LV function on the baseline images, which were suboptimal despite using contrast and led to differences in opinion. It was confirmed that there was no evidence of reversible ischaemia. On one scan, following retrospective reassessment, evidence of reversible ischaemia was present.

Regarding the five false-positive cases, after thorough review, the report changed in three cases to negative for inducible ischaemia. These three cases had been reported with inducible ischaemia localised at the basal inferior and basal infero-septal segments, which can be difficult areas to assess (25).

As per the above-mentioned limitations of the study, we cannot estimate accurately the specificity of the PLDSE service. However, our results have demonstrated that the PLDSE service in our hospital has good predictive value for future cardiac events and, a normal wall-motion response to dobutamine stress was associated with a low incidence of cardiac events.

### Safety of PLDSE service

Safety and tolerability of the DSE has been investigated in numerous studies (26, 27). DSE is considered a relatively well-tolerated diagnostic modality, effective in the management of patients with known or suspected CAD. Adverse effects during testing are relatively frequent, precluding the achievement of a diagnostic end-point in about 5–10% of tests (26). These adverse effects, mostly tachyarrhythmia and arterial hypotension, are usually minor and self-limiting. However, severe life-threatening complications such as acute myocardial infarction, asystole, ventricular fibrillation, sustained ventricular tachycardia or severe symptomatic hypotension, as well as death, have been reported.

In our study, no death, myocardial infarction, ventricular fibrillation or high conduction disturbances were observed. Almost all the incidences of dobutamine adverse effects, apart from two cases, were managed successfully by the physiologist team without the intervention of a physician.

## Conclusions

The current era of cost containment makes it challenging to dedicate physician time solely to the supervision of a time-consuming test such as DSE. In our hospital, the majority of DSE lists are led by physiologists. The innovative practice of independent cardiac physiologist-managed DSE services, without the support of a cardiologist or cardiac nurse for the administration of dobutamine/atropine, appears to be feasible, effective and safe. If this approach were to be adopted by other institutions in the UK, there is significant potential for physiologist-led SE services to improve access to and reduce waiting time for this highly effective diagnostic tool, whilst freeing up specialist medical and nursing staff for other duties. Cardiac physiologists involved in the service are now working towards obtaining the new formal BSE accreditation qualification in SE, which involves sitting an examination, collecting a log book of stress echo cases and by attending a practical exam assessment.

## Supplementary Material

Supporting Table 1

Supporting Table 2

## Declaration of interest

The authors declare that there is no conflict of interest that could be perceived as prejudicing the impartiality of the research reported.

## Funding

This work did not receive any specific grant from any funding agency in the public, commercial, or not-for-profit sector.
