# Supplementary material for: Safety and efficacy of physiologist-led dobutamine stress echocardiography: experience from a tertiary cardiac centre
Source: Echo Res Pract. 2018 Jul 2;5(3):105–12. doi: 10.1530/ERP-18-0038 (PMC6074818; doi:10.1530/ERP-18-0038)
Supplement: Supporting Table 1 [file erp-5-105-t001.pdf]

**Cardiothoracic Directorate**
**The Heart and Lung Centre**
**Department of Cardiac Physiology**

# Prescription for Drug Administration During Stress Echocardiography

(Affix addressograph label here)

NAME:
ADDRESS:
HOSPITAL No:
DATE OF BIRTH: - -/- -/- - - -

Weight (kg):

actual/estimated

ALLERGIES/SENSITIVITIES:

| Compound: | Reaction (eg rash) |
|---|---|
| | |

Signature:
Date:

The following prescription outlines those medications that are to be administered by the cardiac physiologist in accordance with the indications and directions for administration as outlined in Patient Specific Directions. The medications will be administered when performing stress tests for viability/ischaemia using Dobutamine/atropine stress echocardiography protocols.

| Drug | Route of administration | Dose range | Administration | | | | |
|---|---|---|---|---|---|---|---|
| | | | Dose | Date | Time | Signature | |
| Dobutamine 250mg/50ml (standard) | Intravenous injection | 5mcg/kg/min to 40mcg/kg/min | | | | | |
| Dobutamine 250mg/50ml (Aortic stenosis with LVSD) | Intravenous injection | 5mcg/kg/min to 20mcg/kg/min | | | | | |
| Atropine 600mcg/ml | Intravenous injection | 300 – 600mcg Max 1.2mg | | | | | |
| Sulphur hexafluoride (Sonovue) | Intravenous injection | 0.2 to 0.5ml Max 5ml | | | | | |
| Glyceryl trinitrate Oral spray | Oral spray | As Directed 400 to 800mcg | | | | | |
| Sodium chloride 0.9% | Flush | 50ml As directed | | | | | |
| Metoprolol 5mg/5ml | Intravenous injection | 1-2mg Max 15mg | | | | | |

| | |
|---|---|
| Signature of Cardiologist/ Cardiology Registrar/Nurse Prescriber | |
| Name (print): | |
| Date | |

| | |
|---|---|
| Signature of Cardiac Physiologist/Consultant Echocardiographer | |
| Name (print): | |
| Date | |
