# Supplementary material for: Safety and efficacy of physiologist-led dobutamine stress echocardiography: experience from a tertiary cardiac centre
Source: Echo Res Pract. 2018 Jul 2;5(3):105–12. doi: 10.1530/ERP-18-0038 (PMC6074818; doi:10.1530/ERP-18-0038)
Supplement: Supporting Table 2 [file erp-5-105-t002.pdf]

DIRECTIONS FOR DRUGS TO BE ADMINISTERED BY THE CARDIAC PHYSIOLOGIST IN ACCORDANCE WITH THE INDICATIONS AND DIRECTIONS OUTLINED IN PATIENT SPECIFIC DIRECTIONS

The following named patients will be administered medications by a cardiac physiologist when performing stress tests for viability/ischaemia using Dobutamine/Atropine Stress Echocardiography protocols as outlined in Patient Specific Directions

Date of Test:
Name and Contact of Doctor covering list:

Patient's name	Date of birth	Hospital Number

1. Signature of Cardiac Physiologist: ……………………… Date: …………..

Name (print): ……………………………………………………..

2. Signature of Cardiologist/ Cardiology Registrar: ……………..Date: ………

Name (print): ………………………………………………………
